# Combined application of bacteriophages with a competitive exclusion culture and carvacrol with organic acids can reduce *Campylobacter* in primary broiler production

**DOI:** 10.1038/s41598-024-59563-w

**Published:** 2024-04-22

**Authors:** E. Peh, V. Szott, B. Reichelt, A. Friese, M. Ploetz, U. Roesler, S. Kittler

**Affiliations:** 1 Institute for Food Quality and Food Safety, University of Veterinary Medicine Hannover, Foundation, Hannover, Germany; 2 Institute of Food Safety and Food Hygiene, Freie Universität Berlin, Berlin, Germany; 3 Institute for Animal Hygiene and Environmental Health, Freie Universität Berlin, Berlin, Germany

**Keywords:** Microbiology, Medical research

## Abstract

For reducing *Campylobacter* (*C*.) in the food production chain and thus the risk to the consumer, the combined application of different measures as a multiple-hurdle approach is currently under discussion. This is the first study to investigate possible synergistic activities in vivo, aiming at reducing intestinal *C. jejuni* counts by administering (i) bacteriophages (phages) in combination with a competitive exclusion (CE) product and (ii) carvacrol combined with organic acids. The combined application of the two selected phages (*Fletchervirus* phage NCTC 12673 and *Firehammervirus* phage vB_CcM-LmqsCPL1/1) and the CE product significantly reduced *C. jejuni* loads by 1.0 log_10_ in cecal and colonic contents as well as in cloacal swabs at the end of the trial (33 and 34 days post hatch). The proportion of bacterial isolates showing reduced phage susceptibility ranged from 10.9% (isolates from cecal content) to 47.8% (isolates from cloacal swabs 32 days post hatch) for the *Fletchervirus* phage, while all tested isolates remained susceptible to the *Firehammervirus* phage. The use of carvacrol combined with an organic acid blend (sorbic acid, benzoic acid, propionic acid, and acetic acid) significantly reduced *Campylobacter* counts by 1.0 log_10_ in cloacal swabs on day 30 only.

## Introduction

*Campylobacter* (*C.*) is a major cause of bacterial foodborne gastrointestinal infections in humans in the EU and worldwide^1^. Poultry is recognized as the main reservoir for *Campylobacter*, and consumption of contaminated poultry products, especially chicken meat, is responsible for the majority of global campylobacteriosis cases in humans^2^. Thus, alternative intervention strategies that directly target these foodborne pathogens in the poultry chain should be considered to reduce the risk for public health^3^. The European Food Safety Authority (EFSA) estimated that the risk of human campylobacteriosis can be reduced by 58% if the cecal *Campylobacter* loads in chicken are reduced by 3 log_10_^4^.

To successfully combat *Campylobacter* at farm level, it is necessary to prevent and impede introduction into poultry flocks, the transmission, colonization, and to reduce its overall prevalence^5^. Results of previous studies indicate that individual measures may not be sufficient to control *Campylobacter*^6,7^. For this reason, it has been suggested that a combination of multiple strategies might be beneficial^3,8,9^, a new control strategy summarized under the term “multiple-hurdle approach”*.* Multiple measures could be applied in addition to farm biosecurity measures, since different modes of action may allow additive synergistic effects along the meat production chain and increase safety of the final product^3,6,7,10,11^. Measures widely discussed as mitigation strategies against *Campylobacter* in poultry primary production include bacteriophages (phages), competitive exclusion (CE) cultures, organic acids, and plant extracts^3,7^.

Phages are viruses that specifically lyse certain bacterial species or strains. Their specificity represents a major advantage compared to other antimicrobial measures such as antibiotics, as the intestinal microbiome is not affected by phages^12^. CE cultures can be obtained from the intestines of healthy adult animals or any other healthy donor. They consist of non-pathogenic bacteria that can occupy intestinal niches after oral administration and thus, might prevent colonization by pathogenic bacteria^13^. Two previous studies successfully combined phages and CE cultures to reduce *Salmonella* Typhimurium in broilers and demonstrated synergistic effects in one trial where the CE culture was applied via spray^14,15^. However, despite the promising results of single applications of the two methods to reduce *Campylobacter*^13,16–19^, the combined use of phages and CE cultures has not yet been examined for this pathogen.

Carvacrol is a phytochemical found in different plant essential organic oils. The mechanism of action of carvacrol is based on the disruption of the bacterial outer membrane^20^. Similarly, the antibacterial effectiveness of some organic acids, especially medium chain fatty acids, relies on disruption of cell integrity^21–23^. Another mechanism of action, primarily of short chain fatty acids, is that they interfere with various intracellular functions of the bacteria after diffusing through the cell membrane^24–26^. Organic acids and phytochemicals such as carvacrol have been widely studied in the past, but there are only few data on the effect of combined use with other measures. Grilli et al.^26^ demonstrated synergistic effects of using propionic acid, sorbic acid, thymol, and eugenol in vitro. The combined application of that blend in a chicken animal model resulted in *Campylobacter* counts being reduced by up to 5 log_10_ units^26^. Similarly, in an in vitro study conducted by Navarro, et al.^27^, synergistic activities of oregano and lactic acid were shown.

A major hurdle for implementing control measures is the translation of study results from small-scale animal experiments in which, for example, application techniques such as oral administration were used for individual broilers in large broiler flocks comprising thousands of animals. Therefore, choosing a study design resembling natural infection routes and practical settings of broiler production is critical for obtaining data of high practical relevance for *Campylobacter* control.

This study used an innovative study design that reflected field conditions as far as possible to examine carefully selected control measures in a multiple-hurdle approach. Four measures were included that had previously been successfully tested in the same seeder bird study design^13,28–30^. We administered (i) phages in combination with a commercial CE product and (ii) carvacrol in combination with organic acids. For each method, suitable application methods were chosen that were already routinely implemented in poultry production. Administration via spray, drinking water, or feed were tested, based on the characteristics of the substances. In order to mimic dynamics of natural colonization in large broiler flocks, a seeder bird model was used, inoculating only a few broilers and waiting for natural colonization in the remaining flock.

The objective of this study was to investigate possible synergistic effects of different mitigation strategies to control *Campylobacter* colonization in broiler chickens. In order to investigate the underlying mechanisms in more detail, further data were collected on the development of resistance to the phage cocktail applied.

## Results

### All inoculated seeder birds were successfully colonized by *Campylobacter*

Three groups were included in the experiments: a control group, a group treated with phages, and a CE culture (P-CE group) as well as a group treated with carvacrol and organic acids (CR-OA group). Prior to *Campylobacter* inoculation, all three groups of broiler chickens were confirmed to be *Campylobacter* negative by qualitative analysis of cloacal swabs taken four days post hatch (dph).

Seeder birds were orally inoculated 10 dph with 3 × 10^4^ colony forming units (CFU) of the *C. jejuni* target strain. Two days after inoculation (12 dph), all 36 seeders of the groups excreted *C. jejuni.*

### Combined treatment with phages and a complex CE-culture significantly reduced *Campylobacter* loads

**Figure 1:**
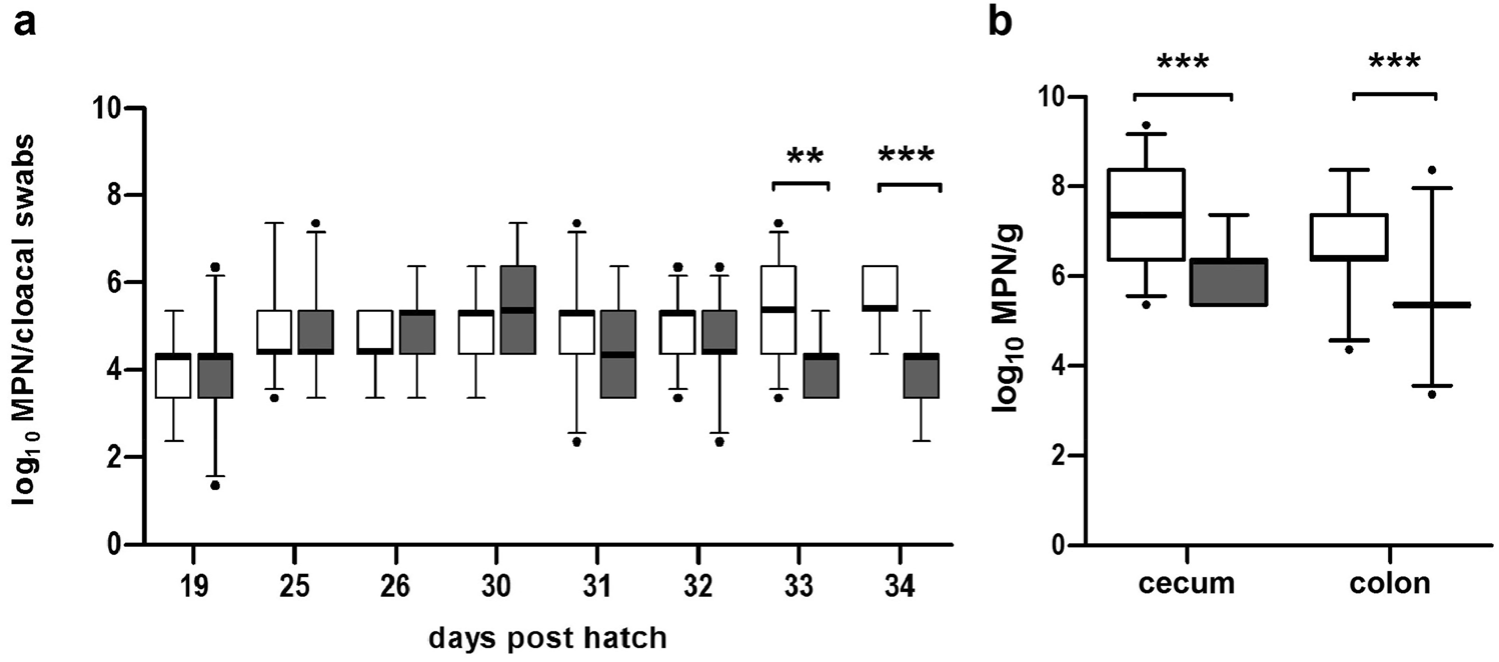
*Campylobacter* (*C*.) *jejuni* concentrations in samples of 23 sentinels per group in the P-CE (phages and competitive exclusion (CE) product) and the control group. Day-old chickens in the P-CE group were sprayed with the CE product one dph (day post hatch) and received the CE product via drinking water 25 dph. Phages were applied via drinking water 30, 31, and 32 dph. (**a**) *Campylobacter* concentrations in log_10_ most probable number (MPN) in cloacal swabs taken from sentinel broilers. Cloacal swabs on 25 dph and 30 dph were taken prior to CE product and first phage application, respectively. (**b**) *Campylobacter* concentrations in log_10_ MPN per gram in cecal and colonic contents of sentinels in the control and P-CE group after dissection (34 dph). Control group (▭); experimental group treated with phages and a CE product (▬). The box plots show the 5^th^ and 95^th^ percentiles (whiskers) and outliers (●). Medians (bold lines) and significance levels (p values) between groups determined by the Mann–Whitney* U* test are shown (*p < 0.05, **p < 0.01, ***p < 0.001).

The results on *Campylobacter* loads of the control group and P-CE group are shown in Fig. 1.

The CE product was applied via spray on the first day of hatch. Fourteen days later (14 dph), 12 sentinels (repeatedly sampled non-inoculated but “naturally colonized” contact animals) of the P-CE group and nine sentinels of the control group excreted *Campylobacter*. On day 19 post hatch, all cloacal swabs taken from sentinels contained *C. jejuni*.

The CE product was applied a second time via drinking water 25 dph. Median *Campylobacter* counts in the P-CE group increased by 1 log_10_ unit from 4.36 log_10_ most probable number (MPN)/cloacal swabs to 5.36 log_10_ MPN/cloacal swabs, while counts in the control group remained stable at 4.36 log_10_ MPN/cloacal swabs. However, the increase in *Campylobacter* counts in the P-CE group was not significant.

Phages were administered continuously via drinking water on 30, 31, and 32 dph. One day after the first phage application (31 dph), median *Campylobacter* concentrations decreased by 1 log_10_ unit to 4.36 log_10_ MPN/cloacal swabs (31, 32, 33, and 34 dph), while the median counts of the control group remained constant at 5.36 log_10_ MPN/cloacal swabs until 34 dph. The difference between the groups was significant 33 and 34 dph (33 dph: *p* < 0.01, *r* = 0.5; 34 dph: *p* < 0.001,* r* = 0.73 (34 dph)).

The analysis of cecal samples 34 dph demonstrated a significant decrease (*p* < 0.0001; r = 0.61) of *C. jejuni* cecal colonization for the P-CE group (*Md* = 6.36 log_10_ MPN/g) compared to the control group (*Md* = 7.36 log_10_ MPN/g). The observed difference in cecal *Campylobacter* counts was 1.0 log_10_ MPN/g. At the same time, *C. jejuni* counts in the colon of P-CE group broilers were significantly reduced (*Md* = 5.36 log_10_ MPN/g; *p* < 0.0001; r = 0.65) by 1.0 log_10_ MPN/g in comparison to broilers in the control group (*Md* = 6.36 log_10_ MPN/g).

### Phage resistance rates were lowest in cecal content

In total, 306 *Campylobacter* isolates were collected prior to, during, and post phage treatment in the P-CE group, and their phage susceptibility was determined. The results are shown in Table 1.

**Table 1:** Proportions of *Campylobacter jejuni* BfR-CA-14430 isolates in the experimental group with reduced phage susceptibility (%).

Phages*	*Campylobacter* isolates with reduced phage susceptibility (%) from						
	Cloacal swabs					Dissection	
	30 dph**	31 dph	32 dph	33 dph	34 dph	Cecum (34 dph)	Colon (34 dph)
NCTC 12673^a^	0.0	26.7	47.8	28.3	26.1	10.9	23.9
LmqsCPL1/1^b^	0.0	0.0	0.0	0.0	0.0	0.0	0.0

*Phages were administered via drinking water 30, 31, and 32 dph (day post hatch).

**Isolates were collected prior to the first phage application.

^a^Reduced plaque counts in overlays by a minimum of 2.4 log_10_ compared with the original *Campylobcater* target strain.

^b^Reduced plaque formation determined by spot testing by a minimum of 2.7 log_10_ compared with the original *Campylobacter* target strain.

All 46 tested *Campylobacter* isolates collected from samples prior to phage application were susceptible to the two applied phages. In samples collected during phage administration (30–32 dph), the percentage of isolates with reduced susceptibility to the *Fletchervirus* phage NCTC 12673 increased to a maximum of 47.8% and then continuously decreased to 26.1% (34 dph) after phage application. The lowest proportion of isolates with reduced susceptibility to phage NCTC 12673 was observed in the cecum (10.9%), while the percentage in the colon was 23.9%.

None of the isolates showed reduced susceptibility to the *Firehammervirus* phage LmqsCPL1/1.

### Phage concentrations were highest in cecal content

**Figure 2:**
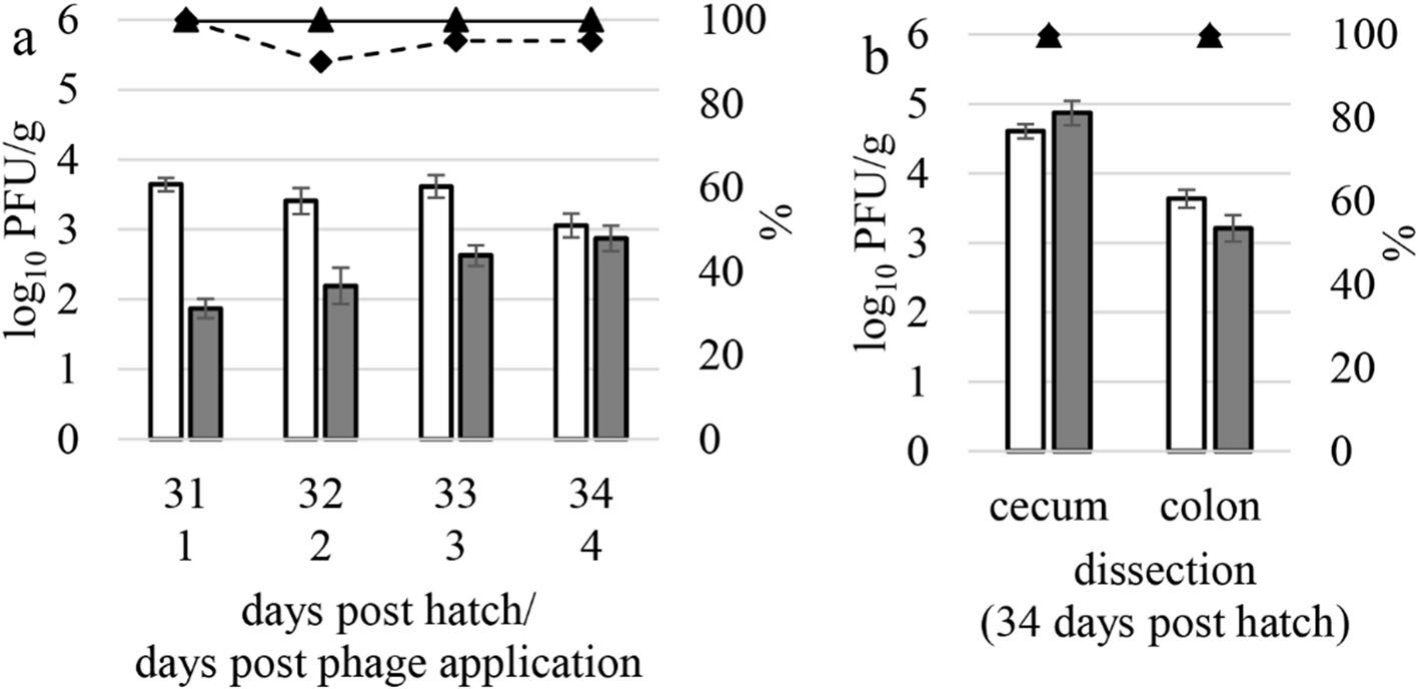
Mean phage concentrations in log_10_ PFU/g and percentage of positive samples (%) in the P-CE group treated with a combination of phages and competitive exclusion (CE) product. Phage counts (**a**) in fecal samples 31, 32, 33, and 34 dph (day post hatch) and (**b**) in cecal and colonic content obtained during dissection 34 dph. White bars represent the phage NCTC 12673; gray bars represent the phage vB_CcM_LmqsCPL1/1 (LmqsCPL1/1). The solid line with triangle represents the phage NCTC 12673 positive samples; the dotted line with diamond represents the phage LmqsCPL1/1 positive samples. Error bars represent standard errors of the mean values.

Per sampling after the first phage application 30 dph, 20 fecal samples and 23 cecal and colonic samples of sentinels, respectively, were obtained and quantitatively analyzed for phages. The results are shown in Fig. 2.

All tested samples contained *Fletchervirus* phage NCTC 12673, and the *Firehammervirus* phage LmqsCPL1/1 detection rate varied between 90 and 100%.

The highest phage concentrations were found in the cecum with a mean concentration of 4.61 log_10_ plaque forming units (PFU)/g for the phage NCTC 12673 and 4.87 log_10_ PFU/g for the phage LmqsCPL1/1.

The concentrations of phage NCTC 12673 in fecal samples and colonic content ranged from 3.06 to 3.64 log_10_ PFU/g. Phage LmqsCPL1/1 concentrations in fecal samples increased from 1.87 log_10_ PFU/g (31 dph) to 2.87 log_10_ PFU/g (34 dph), while the concentration in the colon was 3.21 log_10_ PFU/g (34 dph).

Mean phage concentrations in the drinking water decreased after 24 h in the buckets by 0.95 log_10_ PFU/mL (phage NCTC 12673) and by 0.70 log_10_ PFU/mL (phage LmqsCPL1/1) compared to the initial concentration.

### Combined treatment with carvacrol and organic acids did not consistently reduce *Campylobacter* colonization

Four dph, 13 sentinels in the CR-OA group and nine sentinels in the control group excreted *Campylobacter*. On day 19 post hatch, all sampled sentinels were colonized by *C. jejuni*.

**Figure 3:**
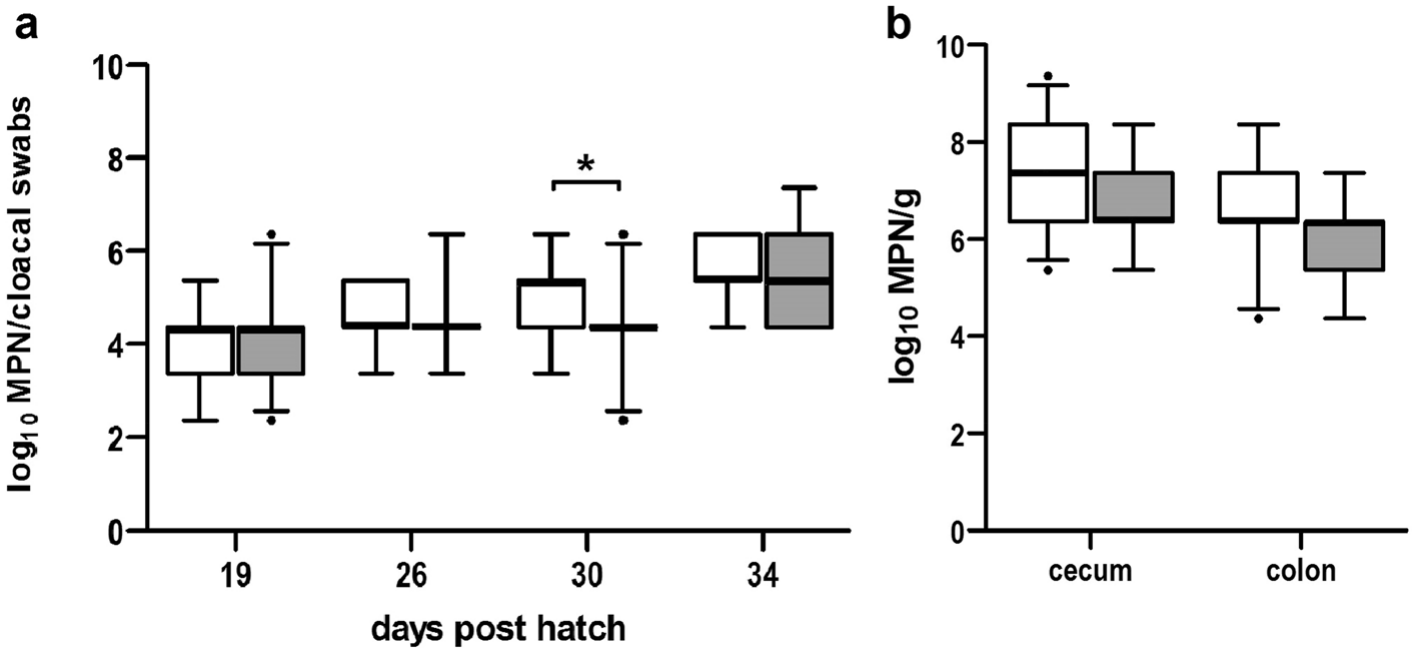
*Campylobacter* concentrations in 23 sentinel samples in the carvacrol/organic acids (CR-OA) group, respectively. Carvacrol was continuously applied via feed and organic acids via drinking water. (**a**) *Campylobacter* concentrations in cloacal swabs taken from sentinel broilers (log_10_ most probable number (MPN)/cloacal swabs). (**b**) *Campylobacter* concentrations in cecal and colonic contents of sentinels after dissection (log_10_ MPN/g) 34 dph. Control group (▭); experimental group treated with carvacrol and organic acids (▬). The box plots show the 5th and 95th percentiles (whiskers) and outliers (●). Medians (bold lines) and significance levels (p values) between groups as determined by the Mann–Whitney* U* test are shown (*p < 0.05, **p < 0.01, ***p < 0.001).

Significantly lower *C. jejuni* counts were found in cloacal swabs of the CR-OA group (*Md* = 4.36 log_10_ MPN/cloacal swabs) compared to the control group (*Md* = 5.36 log_10_ MPN/cloacal swabs) 30 dph (Fig. 3). This corresponded to a reduction of 1.0 log_10_ (*p* < 0.05, *r* = 0.34). No significant differences were observed between *Campylobacter* counts in cloacal swabs of the CR-OA group and the control group 26 and 34 dph (*p* > 0.05).

Likewise, even though *C. jejuni* counts in cecal contents were 1.0 log_10_ lower in the CR-OA group (*Md* = 6.36 log_10_ MPN/g) than in the control group (*Md* = 7.36 log_10_ MPN/g), this reduction was non-significant (*p* > 0.05). The same was true for colonic content, where *Campylobacter* counts did not significantly differ between both groups (*p* > 0.05, *Md* = 6.36 log_10_ MPN/cloacal swabs).

### Applied measures showed no adverse effects on broilers’ growth performance

The treatment showed no effect on the animals’ growth performance. At the end of the trial, no significant differences in the final mean body weight were observed (*p* > 0.05) between broilers in the P-CE group (2.19 kg) or CR-OA group (1.99 kg) compared to broilers in the control group (2.13 kg).

## Discussion

Despite tremendous efforts, effective and easily applicable intervention strategies to effectively control *Campylobacter* on broiler farms are still lacking, thus highlighting the need for further investigations^3,5,31^. In this study, two different multiple-hurdle approaches were tested, combining phages with a CE product and carvacrol with organic acids to evaluate potential synergistic effects.

The animal trial was conducted under field-like conditions to collect data of high relevance for application in commercial broiler fattening. In order to imitate the colonization process of natural *Campylobacter* spreading within a flock, a seeder bird model was used. Only a few animals (seeders) were inoculated with *Campylobacter*, waiting for natural infection of the other birds (sentinels). The dynamic colonization process within poultry flocks can be illustrated only by selectively sampling sentinel birds within the flock and taking high sample sizes that account for the relatively high variance during the colonization process. However, an assignment of fecel samples to seeders or sentinels is not possible without isolating individual animals for defecation. This would interfere with the natural dynamics of the flock and only allows a small number of broilers to be examined. Quantitative analysis also only allows for a limited number of samples to be analyzed due to the time-consuming procedure. Therefore, we chose to use cloacal swabs for sampling and the semi-quantitative method for analysis. These techniques allowed us: (i) to ensure that “naturally” infected sentinels were sampled, (ii) to examine the individual course of each of the 23 sentinels per group at a certain time, and (iii) to include a large sample size in our study to address the higher variance of *Campylobacter* concentrations due to differing colonization times of the seeder bird model. Our choice is supported by results of previous studies in which different sampling techniques (cloacal swabs, carcasses, water) and analysis methods (direct plating, MPN technique, qPCR) were compared^32,33^. In those studies, no statistical differences in the quantification for either the source of isolation or the technique used were observed^32,33^.

The tested measures and combinations thereof were carefully chosen for this study, based on several considerations: first, each single measure had been shown to significantly reduce *C. jejuni* colonization in previous in vivo studies using the same study design^13,28–30^. Second, there are promising study results on *Salmonella* that suggest synergistic activity of phages and CE cultures when used in combination, reducing intestinal *Salmonella* concentrations more than when used individually^14,15^. Third, due to the high host specificity of phages in contrast to antibacterial substances as organic acids or plant extracts such as carvacrol, it can be assumed that phages do not affect the composition of *Campylobacter*-free CE cultures, which could otherwise lead to reduced efficacy. Fourth, different combinations of organic acids and essential oils showed synergistic activities in vitro and significantly reduced intestinal *Campylobacter* concentrations in in vivo broiler studies^26,34,35^. Fifth, the selected combinations can be easily combined even in large broiler stocks, as they are either administered at different time points (phages, CE product) or via different application routes (carvacrol, organic acids). The latter also offers the advantage that two possible environmental sources (feed and water) of *Campylobacter* become decontaminated.

Encouragingly, the combined application of phages and the CE product (P-CE group) significantly reduced *Campylobacter* counts by 1 log_10_ unit in cloacal swabs 33 and 34 dph, and in cecal and colonic contents 34 dph. Since this is the first study testing phages and a CE culture in combination to mitigate *Campylobacter* loads in broilers, no data are available for direct comparison. However, previous studies successfully combined phages and CE cultures to reduce *Salmonella* counts in broilers. Borie et al.^14^ demonstrated synergistic effects of the combined application, which resulted in a reduction in cecal *Salmonella* Enteritidis counts to 1.6 × 10^2^ CFU/g compared with the control group harboring 1.56 × 10^5^ CFU/g, the single application of the CE culture containing 4.23 × 10^3^ CFU/g, and the phage application group showing mean counts of 9.48 × 10^3^ CFU/g *Salmonella* Enteritidis. Similarly, Toro et al.^15^ showed that the combined use of phages and a CE culture significantly reduced *Salmonella* Typhimurium loads, but no synergistic effects were observed compared to a single application. Nonetheless, the study designs and thus the comparability of the studies differed; for example, in the application route, as the measures were applied either as a spray^14^ or orally^15^.

Compared to a previous study using the same CE product as in this study as a single measure^13^, the effect on *Campylobacter* counts was lower at the beginning of the trial. During single application of CE cultures, *Campylobacter* concentrations were significantly reduced by 1 to 2 log_10_ units as early as 18, 21, 26, and 28 dph after use (1 dph, 25 dph)^13^. In contrast, no significant reductions were observed during this period in the present study. Since the phages were administered a few days after the last CE application, antagonistic effects between the two measures cannot be the reason for the differing results. Instead, one possible explanation for the lacking effect of the CE product at the beginning of the animal trial is that it is a non-standardized natural product. The CE product is originally isolated from the cecal microbiota of specific pathogen-free adult broilers and the composition may therefore vary between batches, which may limit the reproducibility of results. Similarly, in a previous study^36^, inconsistent *Campylobacter* reductions were observed after applying a CE culture obtained from chickens. In contrast, in a study by Hakkinen et al., the use of a CE product resulted in a reproducible reduction in the percentage of *Campylobacter* positive broilers and up to 10^9^-fold reduction in *Campylobacter* loads compared to the control group^37^.

It is promising that the effect of combined CE-P application in the ceca was greater than after single application of the same phages, which did not result in significant reductions in cecal *Campylobacter* concentrations as previously published^30^. One possible mechanism of mutual synergistic enhancement could rely on the presence of Lactobacilli. Lactobacilli were shown to reduce *Campylobacter* adhesion to the mucus layer, resulting in free planktonic residence of the bacteria in the intestinal lumen instead of being surrounded by protective mucus^38,39^. The synergistic effect may be further enhanced by the capacity of phages to elevate Lactobacillus populations, as noted by Upadhaya et al.^40^. This might enhance the ability of phages to target and lyse the *C. jejuni* cells. Another possible synergistic action is that phages themselves might reduce *Campylobacter* colonization of the mucus. In a study by Almeida et al.^41^, it was shown that phages used prophylactically bind to the mucosal surface and act as a protective phage layer that prevents colonization of the mucus with *Flavobacterium columnare*. Another possible explanation for the higher effect on cecal *Campylobacter* loads of the combined treatment compared to the single phage use can be suggested when looking at the susceptibility data. The proportions of isolates with reduced phage susceptibility were significantly lower in the combination group P-CE (10.9%) than after single phage application (23.6%). As reported earlier, higher incidences of *Campylobacter* subpopulations with reduced phage susceptibility correlated with lower antibacterial efficacy^17^. The high phage detection rates observed in this study demonstrate that the drinking water is an effective application method that ensures colonization of the chickens with the phages and that phages were able to replicate in the chicken gut.

In contrast to the P-CE group, the combined use of carvacrol and organic acids (CR-OA group) resulted only in a short-term reduction of *Campylobacter* loads. With one exception (30 dph, reduction of 1 log_10_ MPN), no significant reductions in *C. jejuni* concentrations were detected in cloacal swabs, cecal or colonic contents of the sampled chickens. In contrast, single application of the two measures led to significant *Campylobacter* reductions by 1 to 2 log_10_ units in the colon and cecum (carvacrol) and/or in cloacal swabs (carvacrol, organic acids) as previously published^28,29^. The reason for the reduced efficacy of the combined use of carvacrol and organic acids in this study remains unclear. One possible reason for the lacking effect of the combined treatment is that the two measures might have acted antagonistically, thus reducing each other´s efficacy. However, there is no evidence in the literature to support this hypothesis. For example, in a previous in vitro study, synergistic activity of oregano and lactic acid in inhibiting the growth of *Campylobacter* in broth microdilutions was demonstrated^27^. Similarly, Grilli et al.^26^ observed synergistic effects of propionic and sorbic acid, thymol and eugenol in vitro. The combined application of the blend in a chicken animal model resulted in up to 5 log_10_ units reduced *Campylobacter* counts^26^. In addition, Thibodeau et al.^42^ observed in an in vivo study that a combination of microencapsulated organic acids (fumaric acid, sorbic acid) and thymol as an essential oil significantly reduced the intestinal colonization of one *C. jejuni* strain. Nevertheless, colonization by the second tested strain was not reduced in the cited study. This points out the high metabolic and genetic variability in *Campylobacter* strains. The proposed reason for possible synergistic efficacies between certain organic acids and carvacrol is their partially different modes of action. The antibacterial effectiveness of carvacrol, medium chain fatty acids (sorbic acid), and phenolic acids (benzoic acid) was found to be because they destabilize the outer membrane of bacteria^20–23,43–46^. This might facilitate the influx of the short-chain fatty acids such as propionic acid and acetic acid, which have been shown to exhibit their antibacterial effects in the cytoplasm by disrupting various cellular functions which result in bacterial death^21,47,48^. Further studies would be needed to investigate why the two measures in combination did not exert a reducing effect.

## Conclusion

The efficacy of two different multiple-hurdle approaches was evaluated in a study design close to field conditions, providing data of high relevance for possible future applications in conventional broiler flocks. Application of phages and a commercially available CE product significantly reduced fecal excretion and concentrations of *Campylobacter* in the cecum and colon of broilers. In contrast, the combined use of carvacrol and organic acids resulted only in a short-term reduction of *Campylobacter* loads. Further in vitro and in vivo studies might be useful to evaluate the mechanisms of action of the compounds in combination and the reproducibility of the results.

## Materials and methods

### Ethics

This study was carried out in accordance with the National Animal Protection Guidelines and the Guideline for Animal Welfare of the Freie Universität Berlin, Berlin, Germany. The protocol was reviewed and approved by the German Animal Ethics Committee for the Protection of Animals of the Regional Office for Health and Social Affairs Berlin (LAGeSo), Berlin, Germany under the permit number G 0098/18).

### Animal housing

The animal trials were conducted in the experimental animal facilities of the Center for Infection Medicine of the Department for Veterinary Medicine of the Freie Universität Berlin (biosafety level 2, law on genetic engineering). According to a strict implemented hygiene regime, experimental units were cleaned and disinfected and tested for the presence of *Campylobacter* prior to the beginning of the trials, as described previously^13^. To approximate commercial poultry farming, each treatment group was kept in separate barns with litter (1 kg/m^2^) at a stocking density of 39 kg/m^2^. Temperature (automated electric heating), filtered air (ventilation and HEPA filtration of exhaust air), and artificial daylight in the units were controlled throughout the experimental period and adjusted according to the age of the chickens. Water was administered in buckets with nipple drinkers. Broilers had daily access to fresh drinking water (tap water) and feed (conventional three-phase diet as shown in Table 2) ad libitum throughout the entire experimental period.

**Table 2:** Ingredients of the conventional three-phase diet.

Components per kg	Starter diet (0–8 days)	Grower diet (9–26 days)	Finisher diet (27–33 days)
Crude protein (%)	21.5	21.0	20.0
Crude lipids (%)	4.9	6.4	5.5
Crude fiber (%)	2.9	3.4	3.3
Crude ash (%)	5.3	5.1	4.9
ME, kcal/kg	2961.7	2961.7	2961.7
Calcium (%)	0.9	0.8	0.8
Phosphorous (%)	0.6	0.6	0.5
Sodium (%)	0.1	0.1	0.1
Methionine (%)	0.6	0.5	0.5
Lysine (%)	1.3	1.2	1.1

### Animal trials and experimental setup

**Figure 4:**
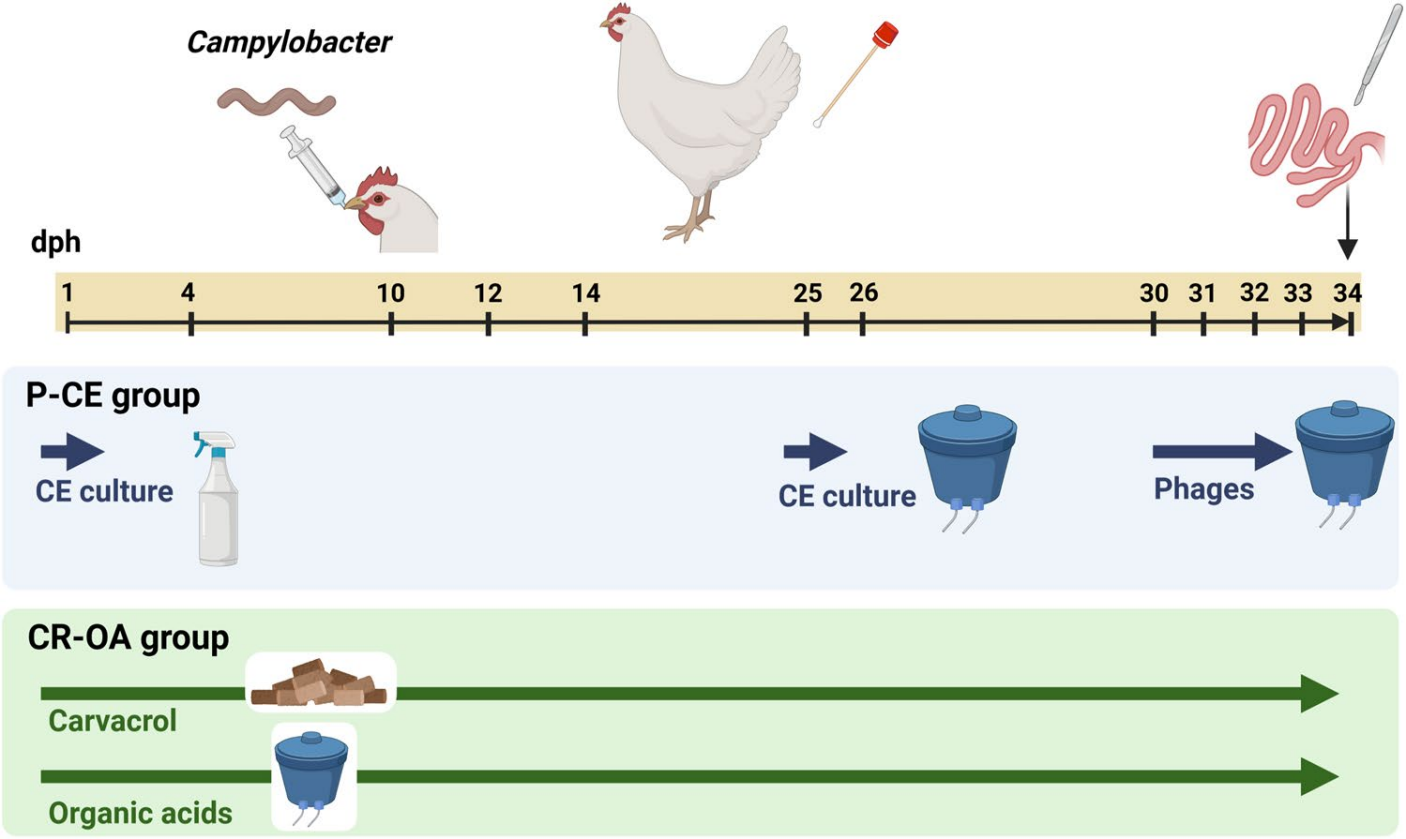
Schematic depiction of the experimental procedure.

For the experiments, a total of 174 eggs of the Ross 308 chicken breed (both aerosols disinfected with formalin and liquid disinfected with WESSOCLEAN^®^ K 50 Gold Line (Wesso AG, Hersbruck, Germany) were obtained from a commercial poultry production facility and incubated for 21 days until hatching. The experimental procedure is shown schematically in Fig. 4. Day-old broilers of both sexes were then randomly assigned to one of the following groups, each group consisting of 58 chickens: control group, CR-OA group (carvacrol and organic acids), and P-CE group (phages and CE product). Broilers were then individually tagged with a unique consecutive number to differentiate between seeders (orally inoculated with the *C. jejuni* target strain on day 10 of age, *n* = 12), sentinels (repeatedly sampled non-inoculated but “naturally colonized” contact animals, *n* = 23), and stocking density broilers (non-inoculated and non-sampled contact animals, *n* = 23). A summary of the measures applied in each group is provided in Table 3.

**Table 3:** Treatment combinations and their application schemes.

Group	Measures	Dose	Application route	Time points of application
Control group	–	–	–	–
P-CE group^1^	Phages	10^7^ PFU/mL	Drinking water	30–32 dph
	CE culture	atm	Spray	1 dph
		atm	Drinking water	25 dph
CR-OA group^2^	Carvacrol	120 mg/kg feed	Feed	1–34 dph
	Organic acids	16 mmol/L	Drinking water	1–34 dph

*atm* according to manufacturer.

^1^Experimental group treated with phages and a complex competitive exclusion (CE) product.

^2^Experimental group treated with carvacrol and organic acids.

Four days post hatch (dph), all 174 broilers were individually monitored for being free of *C. jejuni* by taking cloacal swabs (Sarstedt AG & Co. KG, Nümbrecht, Germany). Seeders were orally challenged with 500 µL Muller-Hinton broth (Oxoid Deutschland GmbH, Wesel, Germany) containing approximately 3.0 × 10^4^ CFU of the *C. jejuni* target strain at the age of 10 days (see below). Thereafter, *C. jejuni* colonization of sentinels was determined following a predetermined sampling scheme. Health and weight gain of the animals were monitored and documented daily. At the end of the experiment, at 34 days of age (average weight 2.0 kg), sentinels were euthanized using ZKS poultry pliers (Corstechnology, Neerstedt, The Netherlands) after confirming deep anesthesia, dissected, and *Campylobacter* counts were determined in cecal and colonic contents.

### *Campylobacter* strains and oral seeder challenge

The whole genome-sequenced *C. jejuni* strain BfR-CA-14430 served as a target strain for the in vivo experiments. The *C. jejuni* strain NCTC 12661 and the *C. coli* field isolate Cc084610 served as host strains for the two phages NCTC 12673 and vB_CcM-LmqsCPL1/1 (LmqsCPL1/1), respectively. The strains were stored at − 80 °C in cryotubes. For recovery, bacteria were spread on Columbia agar plates including 5% sheep blood (Oxoid Deutschland GmbH) and incubated under microaerobic conditions (85% nitrogen, 10% carbon dioxide, 5% oxygen) for 48 h at 37 °C (*C. jejuni* BfR-CA-14430) or 42 °C (*C. jejuni* NCTC 12661 and *C. coli* Cc084610).

For oral seeder challenge, an inoculation dose containing 3.0 × 10^4^ CFU/mL of *C. jejuni* was prepared and seeders were orally challenged into the crop with 500µL of the prepared bacterial suspension, as stated previously^29^.

### Determination of *C. jejuni* loads

Prior to oral inoculation (four dph), cloacal swabs (Sarstedt AG & Co. KG) were taken from all broilers and examined qualitatively for presence of *C. jejuni* in accordance with DIN EN ISO 10272-1:2017-09. Two days post *C. jejuni* inoculation (12 dph), seeders were confirmed to excrete *Campylobacter* by qualitative testing of cloacal swabs.

During the animal experiments, individual *Campylobacter* colonization of sentinels (non-challenged but naturally colonized with *C. jejuni* through contact with seeders) was monitored by semi-quantitative analysis of cloacal swabs. The frequency and timing of the sampling days in the P-CE group and the CR-OA group were chosen, taking into account the different application schemes of the measures in each group. In the control group and the P-CE group, cloacal swabs were collected at the following time points: 19 dph, before and after the second CE culture application (25 and 26 dph), before, during, and after phage application (30, 31, 32, 33 dph), and on the day of dissection (34 dph). Sampling days in the CR-OA group were similar, with the exception that cloacal swabs were not collected on days 25, 31, 32, and 33 post hatch.

Cloacal swabs were taken according to a standardized procedure. The swabs were inserted in the cloaca, rotated five times and excess feces were shaken off. *Campylobacter* analysis was conducted adapted to ISO/TS 10272-3:2010/Cor.1:2011(E), as described earlier^13^. The cloacal swabs were transferred to tubes filled with 3.0 mL Preston broth supplemented with Preston *Campylobacter* selective supplement (SR0117; Oxoid Deutschland GmbH), growth supplement (SR0232; Oxoid Deutschland GmbH), and defibrinated horse blood (SR0050; Oxoid Deutschland GmbH) and homogenized using a vortex shaker (VWR International GmbH, Darmstadt, Germany). A tenfold dilution was prepared in Preston broth and incubated for 24 h at 37 °C under microaerobic conditions. Modified *Campylobacter*-selective charcoal cefoperazone deoxycholate agar (mCCDA) plates prepared from *Campylobacter* blood-free selective agar base (Oxoid Deutschland GmbH) and CCDA selective supplement (Oxoid Deutschland GmbH) were used for *Campylobacter* enumeration. After incubation, dilutions were streaked out on mCCDA plates using 10 µL inoculation loops (Sarstedt AG & Co. KG) and incubated for 48 h at 37 °C in a microaerobic atmosphere. The plates were examined for the presence of *C. jejuni* growth. The highest dilution that showed confirmed bacterial growth was then used to determine the MPN value using an MPN table adapted according to ISO/TS 10272-3:2010/Cor.1:2011(E). Confirmation of suspected colonies was achieved by subculturing onto Columbia agar plates including 5% sheep blood and incubation for 24 h at 37 °C under microaerobic conditions followed by analysis using a Bruker Microflex system for matrix-assisted laser desorption ionization time-of-flight mass spectrometry (MALDI-TOF MS).

To avoid *C. jejuni* or phage cross-contamination between treatment groups, the groups were dissected in batches, starting with the CR-OA group, followed by the control group, and finally the P-CE group. During dissection, intestinal contents from cecum and colon were collected for subsequent *C. jejuni* enumeration. In brief, approximately 1 g of colonic or cecal content was removed aseptically, diluted 1:8 in Preston broth and thoroughly homogenized according to the instructions given in the ISO/TS 10272-3:2010/Cor.1:2011(E). A tenfold serial dilution in Preston broth was prepared and analyzed according to the procedures described above.

### P-CE group: phages

Broilers in the P-CE group received a combination of one *Firehammervirus* phage (LmqsCPL1/1) and one *Fletchervirus* phage (NCTC 12673). The study design, application technique, and concentrations used were the same as those described earlier^30^. Briefly, phages were propagated using the *C. jejuni* strain NCTC 12661 strain (phage NCTC 12673) and the *C. coli* field isolate Cc084610 (phage LmqsCPL1/1). A stock solution was prepared in which the phages each had a concentration of 5.0 × 10^7^ PFU/mL, resulting in a total phage concentration of 1.0 × 10^8^ PFU/mL. The phage blend was diluted 1:10 in tap water (10^7^ PFU/mL) and continuously administered 30, 31, and 32 dph via drinking water. Every 24 h, the drinking water was exchanged and freshly supplemented with phages.

To investigate phage susceptibility of the *C. jejuni* target strain before, during, and after phage application, two colonies per sentinel of the P-CE were picked from mCCDA plates used for determining *C. jejuni* concentrations, The isolates were transferred to tubes filled with 2 mL skimmed milk and stored at − 80 °C. Susceptibility testing was performed as described earlier^30^. Briefly, stock solutions of the phages were prepared with concentrations of 5.0 × 10^3^ PFU/mL (phage LmqsCPL1/1) or 2.5 × 10^3^ PFU/mL (phage NCTC 12673). Phage concentrations were adjusted using the *C. jejuni* target strain BfR-CA-14430. Susceptibility was evaluated using a spot test method (phage LmqsCPL1/1) or the soft-agar overlay method (phage NCTC 12673). For spot testing, 10 µL of the LmqsCPL1/1 phage suspension was spotted on overlay plates. After incubation for 24 h at 37 °C in a microaerobic atmosphere, plaque formation was evaluated. Isolates showing no plaque formation were considered not susceptible to the respective phage.

Phage concentrations in the feces and in cecal and colonic contents as well as phage stability in drinking water were examined in the P-CE group after phage application. For this, 31, 32, 33, and 34 dph, 20 fresh fecal samples per day were collected from the floor and 1 g of each was weighed. In the dissection, 1 g of colonic and cecal contents were collected, respectively. The samples were each transferred to tubes filled with 9 mL SM buffer (5.8 g NaCl, 2.0 g MgSO_4_ × 7H_2_0, 50 mL 1 M Tris, adjusted to pH 7.5, filled up with distilled water to 1000 mL). For assessing phage stability in the drinking water during phage application 30, 31, and 32 dph, water samples were taken immediately after preparation of the phage-supplemented drinking water and 24 h later before water exchange. The samples were filtered using syringe filters with 0.2 µm pore sizes (Carl Roth GmbH + Co. KG, Karlsruhe, Germany). Ten-fold serial dilutions of the samples were prepared in SM buffer. Phage concentrations were quantitatively determined using the soft agar overlay method as described earlier^30^. Briefly, 100 µL of each dilution was mixed with 100 µL of a bacterial suspension of the two phage hosts *C. jejuni* NCTC 12662 (phage NCTC 12673) and *C. coli* Cc084610 (phage LmqsCPL1/1) in molten NZCYM overlay agar (Carl Roth GmbH + Co. KG), 0.7% agar agar). The molten agar was poured onto petri dishes filled with base agar (1.5%). After solidification, plates were incubated for 24 ± 1 h at 41.5 ± 1 °C under microaerobic conditions and plaques were counted to determine phage concentrations.

### P-CE group: complex competitive exclusion product

Broilers in the P-CE group were treated twice with the competitive exclusion (CE) product Aviguard^®^ (Lallemand Animal Nutrition UK Ltd., Malvern, UK) as described in detail earlier^13^. In brief, the broilers received the first treatment by spray on the first day of life and the second treatment on 25 dph via drinking water. After each administration, the CE culture was then confirmed to be free of *Campylobacter*. Briefly, CE culture suspensions were thoroughly homogenized in sterile phosphate-buffered saline (PBS; Oxoid Deutschland GmbH) and examined in serial dilutions plated in 100 µL aliquots on modified *Campylobacter*-selective charcoal cefoperazone deoxycholate agar (mCCDA) plates (CM0739; Oxoid Deutschland GmbH) supplemented with CCDA selective supplement (SR0155; Oxoid Deutschland GmbH). Plates were then incubated in microaerobic atmosphere for 48 h at 37 °C. As specified by the manufacturer, the compound contained the following bacterial species (approximately 10^9^ cells per g): *Escherichia coli, Citrobacter species, Enterococcus species (E. faecalis, E. faecium), Lactobacillus species (L. casei, L. plantarum), Bacteroides species, Clostridium species (C. sporogenes), Eubacterium species, Propionibacterium species, Fusobacterium species, Ruminococcus species*^13^.

### CR-OA group: carvacrol

Broilers of the CR-OA group received experimental diets supplemented with 120 mg/kg feed of carvacrol (Sigma-Aldrich Chemie GmbH, Munich, Germany) with a purity of > 98% as described by Szott et al.^29^. Supplemented feed was provided daily throughout the entire experimental period. To ensure uniform mixing, carvacrol was vaporized in a small amount of feed and then carefully mixed with the rest of the feed. To decrease destabilizing effects, 25.0 kg of the carvacrol-supplemented feed was prepared on demand and stored in airtight containers.

### CR-OA group: organic acids

An organic acid combination of sorbic acid, benzoic acid, propionic acid (Carl Roth GmbH + Co. KG) and acetic acid (E. Merck KG, Darmstadt, Germany) was administered daily to broilers in the CR-OA group in their drinking water, as described previously^28^. Briefly, the mixture of organic acids was administered in a dilution of 1:30 via drinking water to achieve final concentrations of 6.4 mmol/L for sorbic acid, 4.8 mmol/L for benzoic acid, 3.2 mmol/L for propionic acid, and 1.6 mmol/L for acetic acid, resulting in a final organic acid concentration of 16 mmol/L. The drinking water was freshly prepared and changed twice a day. Adding the organic acids to the drinking water lowered the pH of the drinking water to pH 6.0.

### Statistical analysis

Statistical analysis was carried out using SPSS software version 25.0 for Windows (SPSS, Inc., Chicago, IL, USA). Before statistical analysis, individual *Campylobacter* counts were transformed to log_10_ counts and then used as the experimental unit. The Shapiro–Wilk test was used to test the normal distribution of the data. Since our data did not meet criteria of normal distribution, we applied pairwise comparisons using the non-parametric Mann–Whitney *U* test. To ensure alpha error of 0.05, β-error of 0.18, and power of 0.84, 58 animals per group were included in the present study. To determine statistically significant differences, 23 animals were sampled during the animal trial. Probability (*p*)-values below 0.05 were considered statistically significant.

## Data Availability

Data will be made available on request.

## References

[1] Corcionivoschi N, Gundogdu O (2021). Foodborne pathogen *Campylobacter*. Microorganisms.

[2] Authority, E. F. S., Prevention, E. C. f. D. & Control. The European Union One Health 2020 Zoonoses Report. *EFSA J.* **19**, e06971, 10.2903/j.efsa.2021.6971 (2021). 10.2903/j.efsa.2021.6971 PMC9624447 36329690

[3] Soro AB, Whyte P, Bolton DJ, Tiwari BK (2020). Strategies and novel technologies to control *Campylobacter* in the poultry chain: A review. Compr. Rev. Food Sci. Food Saf..

[4] EFSA (2020). Update and review of control options for *Campylobacter* in broilers at primary production. EFSA J..

[5] Abd El-Hack ME (2021). Approaches to prevent and control *Campylobacter* spp. colonization in broiler chickens: A review. Environ. Sci. Pollut. Res. Int..

[6] Kittler S, Shakeri G, Peh E, Plötz M (2021). A One Health perspective on a multi-hurdle approach to combat *Campylobacter* spp. in broiler meat. Curr. Clin. Microbiol. Rep..

[7] Taha-Abdelaziz K (2023). Intervention strategies to control *Campylobacter* at different stages of the food chain. Microorganisms.

[8] Dai L, Sahin O, Grover M, Zhang Q (2020). New and alternative strategies for the prevention, control, and treatment of antibiotic-resistant *Campylobacter*. Transl. Res..

[9] Wales AD, Vidal AB, Davies RH, Rodgers JD (2019). Field interventions against colonization of broilers by *Campylobacter*. Compr. Rev. Food Sci. Food Saf..

[10] Hermans D (2011). *Campylobacter* control in poultry by current intervention measures ineffective: Urgent need for intensified fundamental research. Vet. Microbiol..

[11] Micciche A, Rothrock MJ, Yang Y, Ricke SC (2019). Essential oils as an intervention strategy to reduce *Campylobacter* in poultry production: A Review. Front. Microbiol..

[12] Febvre HP (2019). PHAGE study: Effects of supplemental bacteriophage intake on inflammation and gut microbiota in healthy adults. Nutrients.

[13] Szott V, Reichelt B, Friese A, Roesler U (2022). A complex competitive exclusion culture reduces *Campylobacter jejuni* colonization in broiler chickens at slaughter age *in vivo*. Vet. Sci..

[14] Borie C (2009). Aerosol spray treatment with bacteriophages and competitive exclusion reduces *Salmonella* enteritidis infection in chickens. Avian Dis..

[15] Toro H (2005). Use of bacteriophages in combination with competitive exclusion to reduce *salmonella* from infected chickens. Avian Dis..

[16] El-Shibiny A (2009). Application of a group II *Campylobacter* bacteriophage to reduce strains of *Campylobacter jejuni* and *Campylobacter coli* colonizing broiler chickens. J. Food Prot..

[17] Fischer S, Kittler S, Klein G, Glünder G (2013). Impact of a single phage and a phage cocktail application in broilers on reduction of *Campylobacter jejuni* and development of resistance. PLoS One.

[18] Schoeni JL, Doyle MP (1992). Reduction of *Campylobacter*
*jejuni* colonization of chicks by cecum-colonizing bacteria producing anti-*C*. *jejuni* metabolites. Appl. Environ. Microbiol..

[19] Schoeni JL, Wong AC (1994). Inhibition of *Campylobacter jejuni* colonization in chicks by defined competitive exclusion bacteria. Appl. Environ. Microbiol..

[20] Helander IM (1998). Characterization of the action of selected essential oil components on gram-negative bacteria. J. Agric. Food Chem..

[21] Kim SA, Rhee MS (2013). Marked synergistic bactericidal effects and mode of action of medium-chain fatty acids in combination with organic acids against *Escherichia coli* O157:H7. Appl. Environ. Microbiol..

[22] Desbois AP, Smith VJ (2010). Antibacterial free fatty acids: Activities, mechanisms of action and biotechnological potential. Appl. Microbiol. Biotechnol..

[23] Bergsson G, Arnfinnsson J, Steingrimsson O, Thormar H (2001). Killing of Gram-positive cocci by fatty acids and monoglycerides. APMIS.

[24] Mastromatteo M, Conte A, Del Nobile MA (2010). Combined use of modified atmosphere packaging and natural compounds for food preservation. Food Eng. Rev..

[25] Greenacre EJ, Brocklehurst TF, Waspe CR, Wilson DR, Wilson PDG (2003). *Salmonella enterica* serovar Typhimurium and *Listeria monocytogenes* acid tolerance response induced by organic acids at 20°C: Optimization and modeling. Appl. Environ. Microbiol..

[26] Grilli E (2013). Development of a feed additive to reduce caecal *Campylobacter jejuni* in broilers at slaughter age: From *in vitro* to *in vivo*, a proof of concept. J. Appl. Microbiol..

[27] Navarro M, Stanley R, Cusack A, Sultanbawa Y (2015). Combinations of plant-derived compounds against *Campylobacter in vitro*. J. Appl. Poult. Res..

[28] Szott V (2022). Antimicrobial effect of a drinking water additive comprising four organic acids on *Campylobacter* load in broilers and monitoring of bacterial susceptibility. Poult. Sci..

[29] Szott V, Reichelt B, Alter T, Friese A, Roesler U (2020). *In vivo* efficacy of carvacrol on *Campylobacter jejuni* prevalence in broiler chickens during an entire fattening period. Eur. J. Microbiol. Immunol..

[30] Peh E (2023). Bacteriophage cocktail application for *Campylobacter* mitigation—From *in vitro* to *in vivo*. BMC Microbiol..

[31] Hakeem MJ, Lu X (2020). Survival and control of *Campylobacter* in poultry production environment. Front. Cell Infect. Microbiol..

[32] Rosenquist H, Bengtsson A, Hansen TB (2007). A collaborative study on a Nordic standard protocol for detection and enumeration of thermotolerant *Campylobacter* in food (NMKL 119, 3. Ed., 2007). Int. J. Food Microbiol..

[33] Perdoncini G (2022). Detection and quantification of *Campylobacter* in poultry slaughterhouses using conventional microbiological technique, most probable number, and real-time PCR. Foodborne Pathog. Dis..

[34] Thibodeau A (2015). Chicken caecal microbiome modifications induced by *Campylobacter jejuni* colonization and by a non-antibiotic feed additive. PloS One.

[35] Sima F (2018). A novel natural antimicrobial can reduce the in vitro and in vivo pathogenicity of T6SS positive *Campylobacter jejuni* and *Campylobacter coli* chicken isolates. Front. Microbiol..

[36] Stern NJ (1994). Mucosal competitive exclusion to diminish colonization of chickens by *Campylobacter jejuni*. Poult. Sci..

[37] Hakkinen M, Schneitz C (1999). Efficacy of a commercial competitive exclusion product against *Campylobacter jejuni*. Br. Poult. Sci..

[38] Alemka A, Corcionivoschi N, Bourke B (2012). Defense and adaptation: The complex inter-relationship between *Campylobacter jejuni* and mucus. Front. Cell Infect. Microbiol..

[39] Ganan M (2013). Interaction of *Campylobacter* spp. and human probiotics in chicken intestinal mucus. Zoonoses Public Health.

[40] Upadhaya SD (2021). Bacteriophage cocktail supplementation improves growth performance, gut microbiome and production traits in broiler chickens. J. Anim. Sci. Biotechnol..

[41] Almeida GMF, Laanto E, Ashrafi R, Sundberg LR (2019). Bacteriophage adherence to mucus mediates preventive protection against pathogenic bacteria. mBio.

[42] Thibodeau A (2014). Modification of *Campylobacter jejuni* broiler colonization by a feed additive composed of encapsulated organic acids and essential oils. J. Agric. Sci. Technol. A.

[43] Nowotarska SW, Nowotarski KJ, Friedman M, Situ C (2014). Effect of structure on the interactions between five natural antimicrobial compounds and phospholipids of bacterial cell membrane on model monolayers. Molecules.

[44] Ultee A, Kets EP, Alberda M, Hoekstra FA, Smid EJ (2000). Adaptation of the food-borne pathogen *Bacillus cereus* to carvacrol. Arch. Microbiol..

[45] Ultee A, Bennik MH, Moezelaar R (2002). The phenolic hydroxyl group of carvacrol is essential for action against the food-borne pathogen *Bacillus cereus*. Appl. Environ. Microbiol..

[46] Campos FM (2009). Cell membrane damage induced by phenolic acids on wine lactic acid bacteria. Int. J. Food Microbiol..

[47] Russell JB (1992). Another explanation for the toxicity of fermentation acids at low pH: Anion accumulation versus uncoupling. J. Appl. Bacteriol..

[48] Ricke SC (2003). Perspectives on the use of organic acids and short chain fatty acids as antimicrobials. Poult. Sci..

